# The nature and distribution of affiliative behaviour during exposure to mild threat

**DOI:** 10.1098/rsos.170265

**Published:** 2017-08-09

**Authors:** Guillaume Dezecache, Julie Grèzes, Christoph D. Dahl

**Affiliations:** 1 Institute of Biology, University of Neuchâtel, 2000 Neuchâtel, Switzerland; 2 Laboratoire de Neurosciences Cognitives, Département d'Etudes Cognitives, École Normale Supérieure, INSERM, PSL Research University, 75005, Paris, France

**Keywords:** danger, group, humans, fear, affiliation, grouping

## Abstract

Individual reactions to danger in humans are often characterized as antisocial and self-preservative. Yet, more than 50 years of research have shown that humans often seek social partners and behave prosocially when confronted by danger. This research has relied on *post hoc* verbal reports, which fall short of capturing the more spontaneous reactions to danger and determine their social nature. Real-world responses to danger are difficult to observe, due to their evanescent nature. Here, we took advantage of a series of photographs freely accessible online and provided by a haunted house attraction, which enabled us to examine the more immediate reactions to mild threat. Regarding the nature and structure of affiliative behaviour and their motivational correlates, we were able to analyse the distribution of gripping, a behaviour that could either be linked to self- or other-oriented protection. We found that gripping, an affiliative behaviour, was common, suggestive of the social nature of human immediate reactions to danger. We also found that, while gripping behaviour is quite stable across group sizes, mutual gripping dropped dramatically as group size increases. The fact that mutual gripping disappears when the number of available partners increases suggests that gripping behaviour most probably reflects a self-preservative motivation. We also found age class differences, with younger individuals showing more gripping but receiving little reciprocation. Also, the most exposed individuals received little mutual gripping. Altogether, these results suggest that primary reactions to threat in humans are driven by affiliative tendencies serving self-preservative motives.

## 1. Introduction

How do humans collectively react to danger? The resurgence of terrorist attacks around the world has shown that this question is urgent in today's research agenda. Given their evanescence, immediate reactions to danger are difficult to observe. As a consequence, the exact nature of immediate collective reactions to danger remains to be comprehensively understood. In particular, the question of whether humans prevalently experience social or non-social drives and self-preservative or prosocial motives when being threatened is at the cornerstone of current debates [1].

Although human reactions to danger are commonly characterized as antisocial and self-preservative in nature (an assumption embraced by a large array of audiences [2]), research has shown that affiliation is a main drive during exposure to danger, and prosociality is a common response, even when people's life is directly at risk (see [1] for a review). Behavioural responses in such contexts have been explained by the maintenance of social norms [3,4], the fact that affiliation is a primitive response to danger [5] and the emergence of a social identity among endangered individuals [6]. However, such data are based on *post hoc* reports by and/or interviews from survivors, sometimes obtained years after the disaster took place. If laboratory experiments [7,8] have shown similar affiliation and prosocial responses in yet urged individuals, the ecological validity of those data remain to be corroborated by real-time measures of responses to danger.

In this study, we took advantage of a number of photographs freely released by the Nightmares Fear Factory [9], a haunted house attraction situated in Niagara Falls, Canada. Each photograph represents a group of people (ranging from two to seven individuals) showing spontaneous reactions to a threatening element (of a nature unknown to us) in their environment. Among spontaneous reactions, particularly salient behaviours are hiding behind as well as gripping another person. While hiding clearly is sustained by self-preservative motives, gripping is an affiliative behaviour which might be served by two contradictory motives: gripping could be either produced to protect oneself, or as a mean to protect others. However, the distribution and reciprocity of gripping, we thought, could help shed light on the motivational correlates of this behaviour in threatening contexts. In fact, a low rate of mutual gripping could indicate a prevalence of self-preservative strategies (in particular when the agents is in position to offer mutual protection to a person who is gripping them but do not).

**Figure 1.**
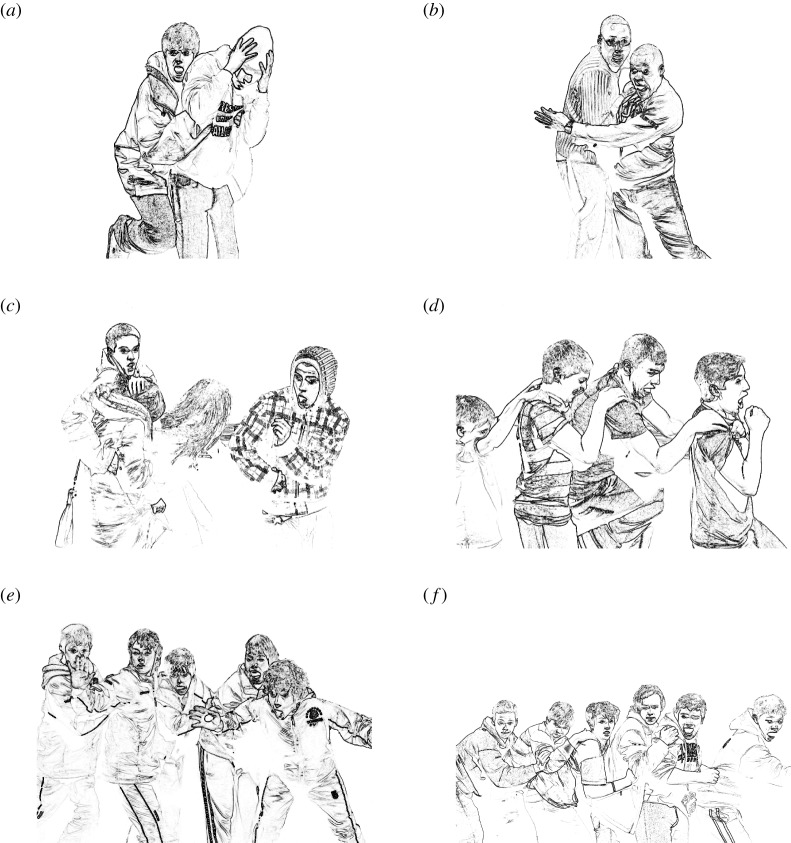
Example of photographs redrawn (note that real photographs were used for coding; they can be found at: https://www.flickr.com/photos/nightmaresfearfactory). (*a*) Showing individual 1 (left) gripping individual 2 (right). (*b*) Showing a mutual gripping between individuals 1 and 2. (*f*) Shows a pattern common in the dataset, the total absence of mutual gripping in a large group. Individual 1 (at the very left) is at the end of the queue.

In this research, we had two hypotheses: first, we hypothesized that primary reactions to danger are affiliative [5,10,11]. Second, since prosociality is known to be preserved in threatening contexts [6,7,12], we should observe more mutual gripping behaviour towards younger and vulnerable individuals. Finally, we also looked at whether fearfulness (the proportion of individuals showing fear) was decreasing with group size, with the hypothesis in mind that grouping is an evolutionary strategy to cope with perceived risk of predation [13]. To examine those hypotheses, we looked at fearfulness, gripping behaviour and mutual gripping in 1293 individuals displayed in 460 scenes, with each scene depicting individuals familiar to one another (see figure 1 for examples). Two blind coders reviewed all the images and coded each individual's (from left (1) to right (*n*)) behaviour, position in the scene and queue (from left (1) to right (*n*), the person at the extreme right being the one closest to the exit or leading the group, as inferred from the group travel direction), sex and age-class.

## 2. Material and methods

### 2.1. Materials and coding

We uploaded photographs from the Nightmare Fear Factory's FlickR account [9] from the years 2012–2014. These photographs are taken during the touring of the attraction, which usually takes 10 to 15 minutes, as specified in the FAQ webpage of the attraction. Group size is supposed to be limited to six individuals (although our coders showed instance of seven individuals together) with groups being strictly composed of individuals familiar to one another. We randomly selected 460 photographs (coders blind to the hypothesis chose them linearly from one folder to another). Two blind coders reviewed all the images and coded each individual's behaviour from left to right. Each individual received a number (from 1 to *n*, from left to right, with *n* being the maximum number of people), was judged for her sex (female or male), her age class (child, juvenile or adult), the total group size (the number of visible individuals in the image, including the individual under coding), whether she expressed fearfulness (presence or absence, as determined by the facial behaviour), whether she was hiding behind others (using others' bodies to hide oneself), whether she was gripping others with one of her hands and how many people she was gripping. Finally, coders counted the number of grips the individual was giving and that were mutual. Two-hundred and sixty data points (circa 20% of the total sample) were then double-coded by a research student. Except for the ‘hiding’ variable (*κ* hiding less than 0), all of the other variables reached strong agreement between judges, as measured by kappa scores (*κ* sex = 0.98; *κ* age class = 0.65; *κ* group size = 0.98; *κ* fearfulness = 0.61; *κ* gripping = 0.84; *κ* number of individuals being gripped = 0.83; *κ* number of grips being mutual = 0.78).

### 2.2. Statistical analysis

Following our hypotheses, we plotted the relative number of people displaying gripping behaviour (general gripping) and the relative number of grips received by a person from a person they are gripping (mutual gripping), and we calculated the ratio of reciprocation by dividing the mutual gripping values by the general gripping values. We then determined linear models, using the least-squares regression approach, that best explained the variations in amount of gripping, mutual gripping or the ratio between general gripping and mutual gripping given the factors ‘sex’, ‘age’, ‘group size’, ‘scene/queue position’ and ‘fear’. We also determined a further linear model to describe the relationship between presence or absence of fear and the group size.

## 3. Results

### 3.1. Gripping behaviour and group size

**Figure 2.**
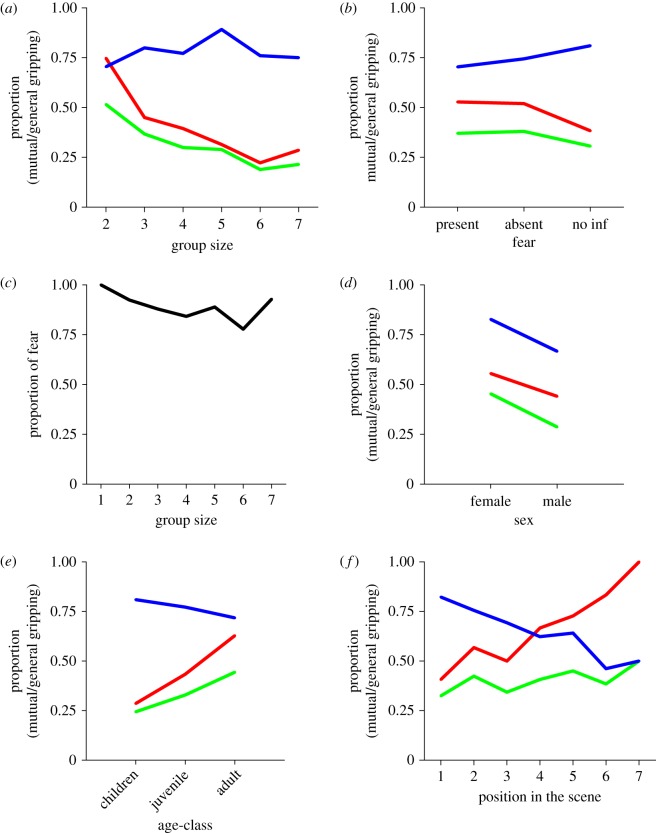
Proportion of gripping (blue line), mutual gripping (green line) and ratio of gripping being mutual (red line) relative to (*a*) social group size, (*b*) presence or absence of fearfulness (no inf = no information), (*d*) sex, (*e*) age-class and (*f*) position in the queue (1 to *n* = from left to right); (*c*) indicates the proportion of individuals showing fearfulness as a function of social group size.

To examine our first hypothesis (that primary reactions to danger are affiliative in nature), we looked at gripping behaviour as a function of group size (figure 2*a*). Gripping behaviour was quantified as (i) general gripping (i.e. the relative number of people displaying gripping behaviour), (ii) received mutual gripping (i.e. the relative number of grips received by a person from a person they are gripping) and (iii) the ratio of the mutual and the general gripping behaviours (gripping ratio). First, we found that gripping was high regardless of group size. We nonetheless found a main effect of group size on general gripping behaviour (*F*_1,1113_ = 15.17, ms = 2.99, *p* < 0.001), as well as on mutual gripping (*F*_1,1118_ = 16.39, ms = 3.61, *p* < 0.001) and the gripping ratio (*F*_1,774_ = 52.33, ms = 11.52, *p* < 0.001).

### 3.2. Gripping behaviour and position in the scene

To examine our second hypothesis (how the distribution of gripping and mutual gripping may help reveal the motivational correlates of gripping), we determined gripping behaviour as a function of position in the scene (1 corresponding to the person at the very left of the scene, the furthest person away from the exit (as inferred from the group travel direction) without anybody behind her and last of the queue) (figure 1*f*). We found a main effect of position on general gripping behaviour (*F*_1,1227_ = 27.27, ms = 38.23, *p* < 0.001) and gripping ratio (*F*_1,861_ = 26.53, ms = 32.75, *p* < 0.001) as well as a trend in mutual gripping (*F*_1,1231_ = 2.81, ms = 3.99, *p* = 0.09). Further the last position in the queue (the first on the scene) was significantly different from all other positions in terms of general gripping behaviour (*F*_1,1227_ = 15.65, ms = 3.56, *p* < 0.001), mutual gripping (*F*_1,1231_ = 6.61, ms = 1.51, *p* < 0.01) and gripping ratio (*F*_1,861_ = 24.79, ms = 5.81, *p* < 0.001), with the highest proportion of gripping compared to other positions in the scene. Interestingly, and as seen in figure 2*f*, proportion of gripping decreases, to reach a minimum at positions #6 and #7, the two leading positions of the group, as inferred from the group travel direction.

### 3.3. Sex-differences in the distribution of gripping and mutual gripping behaviour

As part of the linear model, we calculated the effect of sex (male, female) on the dependent variables (general gripping, mutual gripping and gripping ratio) (figure 1*d*). We found that general gripping behaviour occurs more often in females than in males by showing a main effect of sex (*F*_1,1113_ = 23.11, ms = 4.56, *p* < 0.001). Similarly, mutual gripping and the gripping ratio were affected by sex (mutual gripping: *F*_1,1118_ = 30.66, ms = 6.76, *p* < 0.001; gripping ratio: *F*_1,774_ = 10.95, ms = 2.41, *p* < 0.001), females tending to grip more and to receive more mutual gripping on average than males.

### 3.4. Age class-differences in the distribution of gripping and mutual gripping behaviour

We were further interested in the relationship between gripping behaviour and age classes. As part of the linear model and the *n*-way ANOVA, we used age (child, juvenile, adult) as an independent factor and gripping behaviours (general gripping, mutual gripping and gripping ratio) as the dependent variables. We found that age had no significant effect on general gripping (*F*_1,1113_ = 0.05, ms = 0.01, *p* = 0.82). However, it had an effect on mutual gripping (*F*_1,1118_ = 5.34, ms = 1.18, *p* < 0.05) as well as on the gripping ratio (*F*_1,774_ = 8.41, ms = 1.85, *p* < 0.01). While the proportion of general gripping is stable over all age classes, the proportion of received mutual gripping increases with age-class, indicating that children receive proportionally less grips than adults or juveniles.

### 3.5. Fear and group size

To test the effect of group on group members' expression of fearfulness, we determined the average number of people in a group showing signs of fear (i.e. the proportion showing fear) (figure 1*c*). We statistically compared the proportion of fear across group size and found no effect of group size on proportional fear (*F*_1,1167_ = 0.13, ms = 0.003, *p* = 0.72).

### 3.6. Presence and absence of fear in the distribution of gripping behaviour

The influences of gripping behaviour and/or the presence or absence of fear on the gripping behaviour were investigated. We found no main effect for presence or absence of fear in the distribution of general gripping (*F*_1,1113_ = 0.16, ms = 0.03, *p* = 0.69), mutual gripping (*F*_1,1118_ = 0.001, ms = 0.001, *p* = 0.99), or the gripping ratio (*F*_1,774_ = 0.01, ms = 0.003, *p* = 0.91).

## 4. Discussion

In this research, we took advantage of a set of photographs freely available on the Internet that show individuals familiar to one another and collectively afraid as they are touring a haunted house attraction. We were particularly interested in two hypotheses: first, and against the common belief that reactions to threat are antisocial [2], affiliation is a primary reaction to danger [5]; second, and although reactions to danger are thought to be self-preservative, prosocial motives may prevail under those circumstances. We relied on two consistent behavioural markers, gripping another person, and being gripped back, assuming that high rate of gripping would indicate strong affiliative tendencies in participants, and that high reciprocation in gripping would indicate the maintenance of other-protective strategies. Hiding behind another person could have been a powerful indicator of self-preservative motives but it was proven difficult to reliably identify this behaviour in the present stimuli.

Our first hypothesis was supported: gripping was commonly observed (around 75% of the time), although we found some variations according to age-class and sex.

Our results also showed that, as group size increases, the average number of individuals gripping another remains stable, but mutual gripping decreases dramatically. This reflects that gripping another person (potentially as a mean to protect oneself) is a primeval drive in humans when being confronted by a danger, against our second hypothesis. Mutual gripping, though common when group size is two (when only one other person can be gripped), becomes rare at group sizes of three and more individuals. This suggests that as group size increases, individuals abandon dyadic protection and instead select a strategy of gripping a person who does not necessarily reciprocate the gripping. Those results are suggestive of a core ‘affiliative’ nature of immediate responses to danger [14,15], i.e. that others are seen as a protective figure, as reflected in the stability of the high rate of gripping behaviour in all group sizes.

Comparisons between sex- and age-groups also showed some interesting patterns, with females showing more gripping than males on average, and with children being more likely to grab others. It is known that females tend to adopt a tend-and-befriend strategy when threatened [16,17] instead of relying on a fight-or-flight response. Our results are consistent with this hypothesis, as females tend to rely more strongly on gripping behaviour than males in the study sample. Interestingly, children are the age-class with least recipients of mutual gripping, highly suggestive of a neglect of care for younger individuals in such arousing contexts. This may appear inconsistent with analysis of the Titanic survival records [4] which showed that children had a good rate of survival, probably as the result of the permanence of social norms (women and children were allowed to leave the ship in lifeboats) [18]. Yet, our own analysis was based on more immediate and reflex-like reactions. When time is limited, the prevalence of a social norm favouring children and their mothers may disappear, as seen in the case of the Lusitania sinking [4].

Finally, fear does not become less prevalent as group size increases. One prominent view in the evolution of social living holds that grouping is a response to perceived risk of predation [19] (but see [13] for a critical discussion). Fearfulness (as indexed by the facial expression of the participants) does not seem to be affected by group size in our dataset. This suggests that the potential comfort the presence of others could confer remains limited.

How are those results interpretable in line with what we know from other sources about human collective reactions to danger? Our results first suggest that primary reactions to threat are of an affiliative nature. They also suggest that the distribution of affiliation does not follow a rule of reciprocation, i.e. that others are gripped non-selectively, with the exception of younger individuals receiving little mutual gripping.

Prosociality is known to play a major role during disasters [1,20,21]. One possibility is that prosociality is delayed and intervenes after a more spontaneous self-preservative response. Another possibility is that prosociality only occurs when the nature of the threat has clearly been assessed, a step that may be lacking in the participants we examined. Finally, prosociality might be confused with a tendency to maintain physical contact with others, a pattern that was evident in our dataset.

We see a number of limitations to this study: first, we had few behavioural markers and their link with self-preservative and prosocial motivational components may be difficult to ascertain. However, they were highly reliable and the extent to which gripping is mutual seems to be a strong measure of how much humans finely select their social partners when startled. Second, we only have little information on what happens in this haunted house attraction. Contacting the company running the attraction did not help, as they need to keep some privacy as to what is happening inside the haunted house. In the same vein, it is difficult to tell whether those images are representative of the population since striking images might be more often uploaded, and they might be the ones where people are shown to be more self-preservative in their behaviour. Regarding the very physical structure of the environment of the haunted house, the fact that people tour in the attraction may force them to line up, which may prevent the grabbing of people who are physically more distant. Yet, mutual gripping (e.g. grabbing the person before and after in the line) remains possible and its absence is striking in groups larger than three. Also, we had little information on the relationship between individuals involved in the scenes. The FAQ of the attraction indicates that people are familiar with one another but their exact bond remains impossible to evaluate. This might affect the patterns of gripping, in particular when the scenes involve two individuals only. We could indeed expect small group sizes to be composed of individuals more strongly bonded that those in larger groups, causing a lack of mutual gripping in scenes involving a larger number of persons. As it stands, and provided that people in those scenes were indeed familiar with one another, it remains unknown whether the pattern of responses observed in this study could have been observed in groups of complete strangers visiting the haunted house together.

Despite those limitations, we think that this study is important in that real-time reactions to fear (although mild in our sample) are seldom observed. This is necessary to comprehensively understand the nature and dynamics of immediate collective reactions to imminent danger in humans.

## Data accessibility

Data are available at the Dryad Digital Repository: http://dx.doi.org/10.5061/dryad.r9k5f [22].
